# Supplementary material for: Chlorella pyrenoidosa Polysaccharides as a Prebiotic to Modulate Gut Microbiota: Physicochemical Properties and Fermentation Characteristics In Vitro
Source: Foods. 2022 Mar 1;11(5):725. doi: 10.3390/foods11050725 (PMC8908982; doi:10.3390/foods11050725)
Supplement: Supplementary file 1 [file foods-11-00725-s001.zip › foods-1593986-supplementary.pdf]

Supporting Information

Chlorella Pyrenoidosa Polysaccharides as a Prebiotic to Modulate Gut Microbiota: Physicochemical Properties and Fermentation Characteristics *in Vitro*

Kunling Lv [1,2,†], Qingxia Yuan [2,†], Hong Li [2], Tingting Li [2], Haiqiong Ma [2], Chenghai Gao [2], Siyuan Zhang [1,*], Yonghong Liu [2] and Longyan Zhao [2,*]

[1] College of Light Industry and Food Engineering, Guangxi University, Nanning 530004, P. R. China; kunlinglv@foxmail.com

[2] Institute of Marine Drugs, Guangxi University of Chinese Medicine, Nanning 530200, P. R. China; qingxiayuan@163.com (Q.Y.); hongli12212022@163.com (H.L.); li15578909861@126.com (T.L.); MHQ18878839254@163.com (H.M.); gaochh@gxtcmu.edu.cn (C.G.)

* Correspondence: zhangsiyuangx@sina.com (S.Z.); longyanzhao@gmail.com (L.Z.)

† These authors contributed equally to this work.

Table S1. Content changes of total carbohydrates and reducing sugars during digestion.

Processes	Digestion time (h)	Total carbohydrates (%)	Reducing sugars (%)
Saliva digestion	0	100.00 ± 2.91 [a]	100.00 ± 0.59 [a]
	0.5	102.61 ± 1.94 [a]	101.04 ± 0.59 [a]
	1	106.04 ± 3.30 [a]	101.14 ± 1.61 [a]
	2	102.47 ± 2.91 [a]	99.79 ± 0.59 [a]
Gastric digestion	0	100.00 ± 0.00 [a]	100.00 ± 1.68 [a]
	2	99.67 ± 0.47 [a]	97.63 ± 1.68 [a]
	4	105.34 ± 1.89 [a]	100.00 ± 1.68 [a]
	6	103.67 ± 4.24 [a]	98.82 ± 3.35 [a]
Gastrointestinal digestion	0	100.00 ± 2.21 [a]	100.00 ± 0.46 [a]
	2	100.34 ± 0.16 [a]	99.08 ± 0.69 [a]
	4	102.80 ± 0.47 [a]	100.49 ± 1.15 [a]
	6	101.79 ± 0.32 [a]	99.02 ± 0.92 [a]

In each column, values that do not share a common letter superscript represent significant differences at $p < 0.05$.

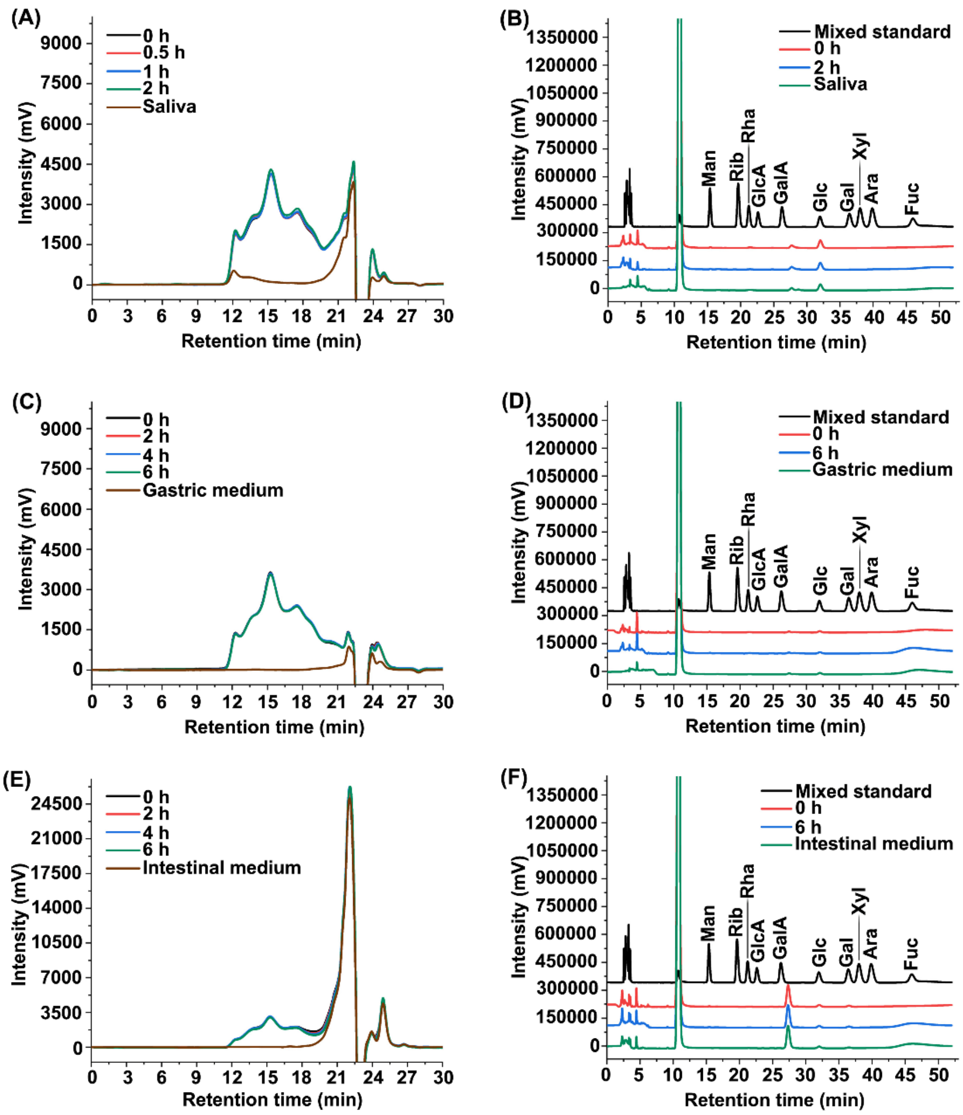

Figure S1. Changes of molecular weight and released free monosaccharides of CPP during simulated digestion (saliva digestion, A & B; gastric digestion, C & D; small intestine digestion, E & F).
